# ACCESS TO NEIGHBORHOOD AMENITIES AND RISK OF INSTITUTIONALIZATION AMONG PERSONS WITH DEMENTIA

**DOI:** 10.1093/geroni/igad104.0165

**Published:** 2023-12-21

**Authors:** Yeon Jin Choi, Gillian Fennell, Jennifer Ailshire

**Affiliations:** University of Southern California, Los Angeles, California, United States; University of Southern California, Los Angeles, California, United States; University of Southern California, Los Angeles, California, United States

## Abstract

Compared to only 2% of the total population between ages 65 and 84, 15% of persons with dementia (PWD) transition to nursing homes. This may be due to caregiving challenges associated with the behavioral symptoms PWD exhibit and their reduced capacities to perform daily tasks. Access to neighborhood amenities (e.g., parks, food access, libraries, service providers) may reduce the risk of institutionalization by helping PWD maintain their health and independence through the provision of opportunities for physical exercise, nutritious diets, social interaction, and cognitive stimulation while providing their caregivers with supportive services. Using data from the2006-2016 waves of the Health and Retirement Study (HRS), the HRS Contextual Data Resource, and the National Neighborhood Data Archive, this study aimed to identify neighborhood factors that are associated with transition into a nursing home among PWD who lived in the community (N=3,366). A series of logistic regression models were estimated adjusting for sociodemographic characteristics, health status, and neighborhood characteristics. Findings suggest that access to more park areas, healthy food outlets (i.e., grocery stores, supermarkets, supercenters), social amenities (e.g., museums, libraries), and social services for older adults and those with disabilities (e.g., senior centers, adult day care centers, disability support groups) were significantly associated with a lower risk of 2-year institutionalization. Retail stores and home health services were not significantly associated with the risk of institutionalization. These findings emphasize the importance of neighborhood amenities, such as parks and healthy food outlets, in enabling PWD to age in place.

